# Research and Remedies

**Published:** 1914-08-01

**Authors:** 


### RESEARCH AND REMEDIES.

### A Curious Dislocation Case.

A very unusual case of double dislocation of the shoulder joints is described in the *South African Medical Record* by Dr. J. J. Levin. One morning he received an urgent message to go to a farm twenty miles away to see a young woman of whom he could learn nothing but that she had been in great pain all night. On arriving at the farm he found a very plump young woman weeping and yelling with pain, and the story he was told was that soon after retiring to bed the previous night she suddenly began to throw herself about and was unconscious for about a minute. From the time of regaining consciousness onwards, she complained of agonising pain round the shoulders. On inspection of the shoulders they appeared of exactly the same outline. Owing to the patient's obesity there was no visible projection of the acromion. Thus at first glance there was nothing to suggest dislocation; and it was only on deep pressure over the joint that Dr. Levin found both glenoid fossæ were empty. In short, the lesion was a double dislocation of the shoulder; each dislocation, moreover, was of the rare subglenoid variety, not the common subcoracoid kind. It was presumed, no doubt correctly, that the attack of unconsciousness was really an epileptic fit, during which the dislocations took place. The author remarks on the difficulty of diagnosis and on the risk of mistaking such a case for a mere hysterical attack, which makes it of interest as a typical difficulty that may arise in general practice.

### A New Leprosy Treatment.

In describing his experience with a novel treatment for leprosy, Dr. B. J. Courtney, of the West African Medical Staff, remarks upon the conflicting reports which have been published of late years upon three or four of the treatments that have been brought forward. Leproline, nastin, chaulmoogra oil, and salvarsan have all been highly eulogised by some authors, and condemned as useless or harmful by others. So, perhaps, it may turn out to be with the method evolved by Dr. Courtney, details of which he publishes in the *Lancet*. This method consists in the intravenous injection of iodoform dissolved in a mixture of paraffin and ether. To start with, half a grain of iodoform is thus administered twice a week; and this is gradually increased to one grain five times a week. Twelve cases were treated, the most inveterate of seventeen, and the most recent of two years' duration. Of the four tubercular cases treated three showed undoubted improvement, and in the fourth the signs were distinctly encouraging at a time when treatment was unavoidably discontinued. In all these cases local injections into the nodules were given, as well as the intravenous injections. In anæsthetic cases no improvement resulted. The author is of opinion that it will be quite worth while to give the remedy a more extended trial and in a larger series of cases, using both intravenous and local injections with due precautions, which are described at length in his paper.

